# Portuguese version of the Literacy Independent Cognitive Assessment (LICA) instrument in the evaluation of individuals aged 50 years or older with Itabaianinha syndrome

**DOI:** 10.20945/2359-4292-2023-0265

**Published:** 2024-08-13

**Authors:** Lucas B. Santos, Michael Kellner, Walter Lisboa, André Faro, Carla R. P. Oliveira, Vanderlan O. Batista, Alécia A. Oliveira-Santos, Íris de Vita Alves de Brito, Cindi G. Marinho, Viviane C. Campos, Keila R. Villar-Gouy, Ângela C. Leal, Rivia S. Amorim, Enaldo V. Melo, Elenilde G. Santos, Roberto Salvatori, Manuel H. Aguiar-Oliveira

**Affiliations:** 1 Divisão de Endocrinologia Programa de Pós-graduação em Ciências da Saúde Universidade Federal de Sergipe Aracaju SE Brasil Divisão de Endocrinologia, Programa de Pós-graduação em Ciências da Saúde, Universidade Federal de Sergipe, Aracaju, SE, Brasil; 2 Department of Psychiatry and Psychotherapy University Hospital Hamburg-Eppendorf Hamburg Germany Department of Psychiatry and Psychotherapy, University Hospital Hamburg-Eppendorf, Hamburg, Germany and Department of Psychiatry and Psychotherapy, Technical University of Munich, Munich, Germany; Department of Psychiatry and Psychotherapy Technical University of Munich Munich Germany; 3 Departamento de Psicologia Universidade Federal de Sergipe São Cristovão SE Brasil Departamento de Psicologia, Universidade Federal de Sergipe, São Cristovão, SE, Brasil; 4 Programa de Pós-graduação em Psicologia Universidade Federal de Sergipe São Cristovão SE Brasil Programa de Pós-graduação em Psicologia, Universidade Federal de Sergipe, São Cristovão, SE, Brasil; 5 Divisão de Psiquiatria Programa de Pós-graduação em Ciências da Saúde Universidade Federal de Sergipe Aracaju SE Brasil Divisão de Psiquiatria, Programa de Pós-graduação em Ciências da Saúde, Universidade Federal de Sergipe, Aracaju, SE, Brasil; 6 Divisão de Geriatria Programa de Pós-graduação em Ciências da Saúde Universidade Federal de Sergipe Aracaju SE Brasil Divisão de Geriatria, Programa de Pós-graduação em Ciências da Saúde, Universidade Federal de Sergipe, Aracaju, SE, Brasil; 7 Divisão de Estatística Programa de Pós-graduação em Ciências da Saúde Universidade Federal de Sergipe Aracaju SE Brasil Divisão de Estatística, Programa de Pós-graduação em Ciências da Saúde, Universidade Federal de Sergipe, Aracaju, SE, Brasil; 8 Division of Endocrinology, Diabetes and Metabolism Department of Medicine The Johns Hopkins University School of Medicine Baltimore Maryland USA Division of Endocrinology, Diabetes and Metabolism, Department of Medicine, The Johns Hopkins University School of Medicine, Baltimore, Maryland, USA

**Keywords:** Illiteracy, dementia, test, translation into Portuguese, growth hormone

## Abstract

**Objectives:**

Individuals with congenital isolated growth hormone deficiency (IGHD) in Northeastern Brazil have a normal lifespan with a prolonged healthspan. We hypothesize that their increased healthspan is accompanied by a reduced cognitive decline during aging. We have recently shown that these individuals have a similar total cognitive function and better attention and executive function than controls. These data were obtained using a Portuguese version of the Literacy Independent Cognitive Assessment (LICA) instrument, whose translation to facilitate cognitive research in Portuguese-speaking countries is described here.

**Subjects and methods:**

In the first stage, a psychologist and a psychiatrist translated the LICA instrument from English into Portuguese, and an English teacher proofread the translation. The second stage included its synthesis and cultural adaptation, carried out by Brazilian authors, and changes in some words and images. The third stage involved an evaluation round with two referees (independent psychologists). The fourth stage involved a back translation of the instrument, which demonstrated > 95% agreement with the original version. The fifth stage included a study to verify the understanding of the questionnaire by responders. In the sixth stage, an endocrinologist and a psychiatrist approved the final Portuguese version of the instrument, which was then administered to 15 individuals with IGHD and 15 controls older than 50 years.

**Results:**

The LICA instrument was applied 59 times (5 times in the pilot study, 24 in the variability studies, and 30 in the experimental step). The interobserver and intraobserver variabilities were 99% and 96%, respectively. Cronbach’s alpha was 0.76, indicating good reliability. The mean (± standard deviation) duration of the application was 39 ± 8.6 and 48.5 ± 5.8 minutes in literate and illiterate individuals, respectively.

**Conclusion:**

The Portuguese version of the LICA instrument was valuable for the cognitive assessment of individuals with Itabaianinha syndrome.

## INTRODUCTION

By the end of this century, there will be 500 million Portuguese speakers in nine countries worldwide. The illiteracy rate in these countries remains high, with Portugal having the lowest rate (5%), followed by Brazil (11.2%), while Angola (58%) and Guinea-Bissau (63%) have the highest rates (1). High levels of education and social involvement are protective factors against dementia and are proportionally inverse to cognitive impairment (2). Illiteracy, *per se*, has been independently associated with a high risk of prevalent and incident dementia in Northern Manhattan, New York (3). In Brazil, two different studies (4,5) have confirmed this relationship. In the most recent study, nearly two-thirds of the individuals considered to have cognitive impairment had no formal education (5).

Population aging has become a worldwide phenomenon, with projections that the number of older people will reach approximately 1 billion by 2030 (5,6). Developing countries (such as most Portuguese-speaking countries) will see the greatest increase in the absolute number of older people, and dementia disorders will pose enormous challenges to public health in these countries (6,7). For example, between 2000 and 2010 in Brazil, the population increased by 12.3%, and the number of people aged 60 years or older increased by 41.6%. About 1 million people are estimated to have dementia in Brazil, of whom 77% are undiagnosed (8).

Several tools have been used to standardize the diagnostic criteria for Alzheimer’s disease and mild cognitive impairment after implementation of the Mini-Mental State Examination (9), which were revised in the Diagnostic and Statistical Manual of Mental Disorders, Fifth Edition, Text Revision (DSM-5-TR) (10). These diagnostic tools include the NINCDS-ADRDA criteria for Alzheimer’s disease, the Alzheimer’s Disease Assessment Scale – Cognitive Subscale (ADAS-Cog), the Consortium to Establish a Registry for Alzheimer’s Disease (CERAD), the Montreal Cognitive Assessment (MoCA), and the Cambridge Mental Disorders of the Elderly Examination (CAMDEX) (11-16). These tools include many items that are appropriate for individuals who can write and read; however, their application to individuals who are illiterate and have low levels of education is questionable.

To overcome this problem, the Literacy Independent Cognitive Assessment (LICA) instrument was developed in South Korea (17-20). It has been used to screen for dementia and mild cognitive impairment in older individuals who are illiterate. The LICA instrument can be universally applied and is potentially useful in developing countries, including many Portuguese-speaking nations.

We have studied extensively for 30 years a cohort of individuals with congenital isolated growth hormone deficiency (IGHD) (21) in the city of Itabaianinha, Brazil (22-25). These individuals have a normal lifespan (26) with an extended healthspan, *i.e.*, the period of life free from major chronic clinical diseases and disabilities (23). The cohort currently includes 15 individuals with IGHD aged 50 years or older, most of whom have low levels of education. This group of individuals offers a unique opportunity for a study of brain aging in untreated congenital IGHD. We hypothesize that their increased healthspan is accompanied by a reduced cognitive decline with aging.

Based on these considerations, the aim of this study was to translate the LICA instrument into Brazilian Portuguese and adapt it to the Brazilian population with the aim of offering this translated and adapted version to researchers in various areas who need to assess the cognitive function of older individuals with low education levels in Brazil and – with minor adaptations – other Portuguese-speaking countries. This study, born from an endocrinological need, exemplifies a transdisciplinary approach that integrates natural, social, and health (endocrinology, nutrition, geriatrics, psychiatry, and neurology) sciences with humanistic tools, as currently recommended in health research (27,28).

## SUBJECTS AND METHODS

### The original version of the Literacy Independent Cognitive Assessment Instrument

The LICA instrument is a 300-point test comprising 13 subtests and 187 questions (17, 18 19, 20). It begins with a literacy assessment section and is followed by 13 sections assessing cognitive domains.

In this article, words in Portuguese or Korean are presented in italics, while those copied from the LICA manual are enclosed in quotes.

#### Literacy assessment section (patient assessment)

The patient is instructed to read two sentences describing the beginning of a story (Supplementary Material 1, page 3). Patients who are able to read both sentences are asked to write a sentence describing what will happen next in the story. If the patient can read and write the sentences, the assessment is interrupted, otherwise, the patient is asked to read and write each word.

#### Literacy assessment section (caretaker’s assessment)

The examiner asks the patient’s caretaker about the patient’s reading and writing ability prior to the illness, using the following questions:

• Writing: **“**Before the illness, was he/she able to write his/her own sentences?” (If “yes”, 3 points; if “no”, the subsequent questions are asked). “But could he/she write words?” (“yes”, 2 points; “no”, 1 point).
• Reading: “Before the illness, was he/she able to read and understand sentences without help?” (If “yes”, 3 points; if “no”, the subsequent questions are asked). “But could he/she read and understand words?” (“yes”, 2 points, “no”, 1 point).

The scoring ranges from 1 (insufficient/failure) to 2 (success in reading and writing three words) and 3 (success in reading and writing the sentences). A patient with a reading and writing score of 3 is considered literate. In all other cases, the patient is considered illiterate.

This section is used solely to assess the patient’s literacy. Its score does not count toward the total LICA score.

#### Cognitive Domains (13 Sections)

The evaluation of the cognitive domains comprises the following sections (or subtests):

• Memory
  -Verbal memory: Story Recall (Subtests 1, 7, and 8) and Word Recall (Subtests 3, 10, and 11)
  -Visual memory: Visual Recognition (Subtest 9)
• Visuospatial Construction: Stick Construction (Subtest 2)
• Language (semantics): Color and Object Recognition (Subtest 13) and Naming (Subtest 13)
• Executive Function: Digit Stroop (Subtest 5) and Word Fluency – Animal Fluency (Subtest 12)
• Attention: Visuospatial Span (Subtest 4)
• Calculation: Calculation (Subtest 6)

Below is the description of each of these subtests.

**Subtest 1: Story Recall – Immediate Recall**

In this subtest, the examiner tells the following story about a student helping an elderly woman: “Kicheol / a middle school student / came across / one / elderly lady / in front of Seoul Station. (2-second interval) The elderly lady / was going / to her daughter’s / house / carrying / a pack / of eggs / and a jar / of red pepper paste. (2-second interval) Kicheol / carried / the jar / of red pepper paste / to the bus station. (2-second interval)”. The patient is then invited to retell the story, which is evaluated in 20 items that are parts of excerpts from the story. This subtest receives a score of 1 point if the items are properly recalled, 0.5 point if only partially recalled, or 0 points if the items are forgotten or inappropriately recalled (Table 1).

**Table 1:** Examples of scoring in the Story Recall – Immediate Recall subtest

Story	Criteria	0.0 Points	0.5 Point	1.0 Point
Kicheol	Kicheol	Cheolsool		Kicheol
A middle school student	Middle school	Elementary school		Middle school
Came across	To meet		Saw	Came across/Met
One	One person	Two people		One (person)
Elderly lady	Elderly lady	Elderly man	Elderly person	Woman
Seoul Station	Seoul Station	Subway station	Station	Seoul station
The elderly lady	Elderly lady	Elderly lady	Elderly person	Elderly woman
Was going	Going	Went/left	Was going to go	Was going
Her daughter’s	Daughter	Cousin/niece	Family	Daughter
House	To the house	To the shop	To her apartment	To the house
Carrying	To carry	On the head		Holding
A pack	A pack		A bag	A pack/ a carton
Of eggs	Eggs	Ball		Eggs
And a jar	A jar	A bag		A jar
Red pepper paste	Red pepper paste	Miso paste		Red pepper paste
Kicheol	Kicheol	Cheolsoo		Kicheol
Carried	Carried	Held the jar together with		Carried
The jar	A jar	A pack		A jar
Of red pepper paste	Red pepper paste	Miso paste		Red pepper paste
The bus station	Bus station	Taxi station	To the bus ride	To the bus stop

**Subtest 2: Visuospatial Construction – Stick Construction**

In this subtest, the patient is given four sticks, each with one end painted red. The patient is then shown 10 images, each with a different shape, and is asked to replicate the shapes using the sticks. A score of 1 point is given if both the shape and the position of the red dots are replicated correctly, 0.5 point if the shape is correct but the red dots are positioned incorrectly, and 0 points if the shape is incorrect.

**Subtest 3: Word Recall – Immediate Recall**

The examiner reads 10 words and instructs the patient to recall as many words as possible. The examiner repeats the test three times, following the same word order, asking the patient to recall the words each time. Each correct word recalled receives a score of 1 point.

**Subtest 4: Visuospatial Span**

The examiner shows a set of blocks to the patient. The examiner points to the blocks in a specific order and asks the patient to point to the same blocks, first in the same order and then in reverse order. If the patient is successful on the first attempt, the examiner considers the second attempt also correct and moves on to the next subtest. If the patient fails the first attempt, the examiner gives the instruction to move on to the second attempt. If the patient fails both attempts, the test is interrupted. The test score is the highest number of blocks (span score) the subject successfully replicates at least once.

**Subtest 5: Digit Stroop**

The Digit Stroop is a two-part test. The examiner presents to the patient a Digit Stroop stimulus plate, which contains a table with 5 columns and 10 rows, with 50 cells in total. During part 1, the subject reads the number written in each cell, and in part 2, counts the number of digits written in each cell. The time limit for each part is 3 minutes. The number of correct and incorrect answers is recorded, as is the time taken for the task. If the task is not completed within 3 minutes, the test is interrupted. The score is calculated by subtracting the execution duration of part 1 from that of part 2 and counting the number of correct answers in part 2.

**Subtest 6: Calculation**

The calculation involves addition, subtraction, multiplication, and division tasks, with questions gradually increasing in difficulty as the test progresses. The patient listens to a question and calculates the answer without using pen and paper. There are two trials for each difficulty level. The examiner reads out the question, and the patient calculates and reports the answer. If the patient succeeds in answering the first question of one difficulty level, the second trial should be considered automatically correct, and the patient should proceed to the next difficulty level. If the patient fails both trials at the same difficulty level, the test should be interrupted and moved to the next section. Each correct answer receives a score of 1 point, and each incorrect answer receives a score of 0 points.

**Subtest 7: Story Recall – Delayed Recall**

In this subtest, the patient is asked to retell the story presented in Subtest 1. The same criteria used for scoring Subtest 1 apply to this subtest.

**Subtest 8: Recognition**

The examiner asks 10 multiple-choice questions with three possible answers about the “story of the student who helped the elderly woman”. The patient is instructed to select the answers that best match the story. Each correct answer receives a score of 1 point, and each incorrect answer receives a score of 0 points.

**Subtest 9: Visual Memory – Visual Recognition**

This delayed recognition test assesses the patient’s ability to recognize, among 20 images, the 10 images presented during the Stick Construction Subtest. Each correct answer receives a score of 1 point, and each incorrect answer receives a score of 0 points.

**Subtest 10: Word Recall – Delayed Recall**

In this subtest, which analyzes delayed recall, the patient is asked to repeat the words presented in Subtest 3.

**Subtest 11: Word Recall – Recognition**

This subtest involves the presentation of 20 words, of which 10 are from the previous subtest and the other 10 are not (incorrect answers). The patient must say “yes” if the word was mentioned in the previous subtest and “no” if otherwise. Each correct answer receives a score of 1 point.

**Subtest 12: Word Fluency – Animal Fluency**

The patient is asked to name as many animals as possible in 1 minute. If the patient remains silent for more than 15 seconds, the examiner repeats the instructions and provides encouragement. The total score in this subtest is the total number of acceptable animal names that the patient reports in 1 minute.

**Subtest 13: Color and Object Recognition/Naming**

The patient is shown an image with two objects. One is a real object, while the other is the original object with some of its features modified. The patient is asked to choose the real object and report the name of the object. The correct identification of the object and its color, or the correct name of the object, receives each a score of 1 point.

#### Total score calculation

To allocate each cognitive domain with an appropriate score, the raw scores were modified to provide a scoring system with a range of 0-300 points (Table 2). The following points are allocated to each domain: memory tests (Story Recall, Word Recall, Visual Recognition), 150 points (50%); visuospatial construction (Stick Construction), 30 points (10%); language (Color and Object Recognition/Naming), 45 points (15%); executive function (Animal Fluency, Digit Stroop), 47 points (15.7%); attention (Visuospatial Span), 16 points (5.3%); and calculation, 12 points (4%). Animal fluency has a maximum score of 22 points, and raw scores exceeding 22 points are assigned 22 points. The examiner marks the patient’s responses and subtest scores on a recording sheet in the scoring program to calculate the converted scores and the total score. Scores below 186.0 and 154.5 define dementia in literate and illiterate individuals, respectively (17-20).

**Table 2:** Subtest converted score for the total LICA score calculation

	Subtest	Raw score range	Converted score range for the total LICA score calculation
1	Story Recall – Immediate Recall	0-20	0-20
2	Stick Construction	0-10	0-30
3	Word Recall – Immediate Recall	0-30	0-20
4	Visuospatial Span	0-16	0-16
5	Digit Stroop	0-25	0-25
6	Calculation	0-24	0-12
7	Story Recall – Delayed Recall	0-20	0-20
8	Story Recall – Recognition	0-10	0-10
9	Visual Recognition	0-20	0-40
10	Word Recall – Delayed Recall	0-10	0-20
11	Word Recall – Recognition	0-20	0-20
12	Word Fluency – Animal Fluency	0-infinity	0-22
13	Color and Object Recognition/Naming	0-15 0-15	0-15 0-30

#### Application duration

The application duration of the Korean version of the LICA instrument is 28.6 ± 6.4 minutes among literate normal individuals and 30.7 ± 5.8 minutes among illiterate individuals (17).

## Preliminary steps taken for the translation of the instrument into Portuguese

The first step in the process of translating and adapting the LICA instrument into Portuguese was to obtain authorization for its use from Inpsyt, Inc., Seoul, South Korea. The person in charge (Mrs. Mirae Park) provided the LICA manual and physical materials upon a token payment of USD 200. Subsequently, the Brazilian team met to define the attributions of the team, which included two psychiatrists with experience in psychiatric research (M.K. and V.O.B.), two psychologists who work with psychological assessment (A.F. and W.L.), one geriatrician with experience in dementia disorders (R.S.A.), one nutritionist who suggested Brazilian foods (A.A.O.S.), nine doctors with long-time experience in conducting research with the Itabaianinha IGHD cohort (L.B.S*.* C.R.P.O., C.G.M., V.C.C., K.K.V.-G., A.C.L., E.G.S., R.S., and M.H.A.-O.), two physicians with expertise in growth hormone research (L.B.S. and M.H.A.O.), one English teacher with two certifications (Cambridge English Level 5 Certificate in Teaching English to Speakers of Other Languages [CELTA] and Certificate in Advanced English [CAE]) with experience in translations (I.V.A.B.), and one statistician (E.V.M.).

## Translation of the instrument

The guidelines for cross-cultural adaptation of self-report measures by Beaton and cols. (29) were followed in six stages. In the first stage, a psychologist and a psychiatrist translated the instrument from English into Portuguese, preserving the meanings and structure of the original version. An English teacher proofread the translation. The second stage included the synthesis and cultural adaptation of the translated version, performed by all the authors, including a nutritionist who proposed common food terms in Portuguese, resulting in a second consensus version in which some words and images were changed. The third stage involved an evaluation round with two referees (independent psychologists), who approved the new version. The fourth stage involved a back translation process, carried out by three researchers in Psychology and Medicine who are bilingual and were unaware of the original questionnaire. This back-translated version reached a compatibility level above 95% in terms of semantic and grammatical equivalence, demonstrating a high level of agreement. The only discrepancies were regarding words that do not have a direct translation into Portuguese or are not part of the Brazilian culture, such as “red pepper paste” and “middle school”. A final meeting adjusted these few specific terms in a consensus version. Therefore, the final version demonstrated 100% agreement with the original Korean version. In the fifth stage, a pre-test was carried out with five individuals who were illiterate and who easily understood the instrument. In the sixth stage, two senior authors – a psychiatrist and an endocrinologist (M.K. and M.H.A.O.) – audited all the steps of the process, assuming that adequate translation had been achieved. This version was subsequently administered to 15 people with IGHD (6 men, age range 53-84 years, 5 of whom were illiterate) and 15 local individuals (controls) with normal height (6 men, age range 56-78 years, 5 of whom were illiterate). Both groups had similar years of schooling (mean ± standard deviation 6.4 ± 5.5 years in the IGHD group and 4.7 ± 4.1 years in the control group). In all, the Portuguese version of the LICA questionnaire was applied 59 times, including 5 in the pilot study, 24 in the variability studies, and 30 in the experimental step.

## Variability and reliability

To assess interobserver variability, the LICA questionnaire was applied by two trained investigators (L.B.S. and V.O.B.) to six individuals, one week apart. To assess intraobserver variability, the questionnaire was applied by a trained investigator (V.O.B.) to six other individuals, also with an interval of 1 week. Variability was calculated by the average percentage of variation in the six pairs of measurements in each case. The test score reliability was calculated using Cronbach’s alpha coefficient.

## Ethical aspects

The protocol of the study was approved by the Institutional Review Board of the Federal University of Sergipe (CEP/UFS) under the number 3.423.043, CAAE 14383319.5.0000.5546.

All participants signed an informed consent form.

## RESULTS

Three words in the literacy assessment (umbrella, picture, and song) were replaced. The single English word umbrella was replaced with the single Portuguese word *cachorro* (dog), as the Portuguese translation of umbrella is a compound word (*guarda-chuva*), which could influence the difficulty of reading and writing it. We also changed the Korean proper name “*Younghee”* for *Carlos,* a very common first name in Portuguese. Therefore, the two translated sentences were “*Carlos estava com muita sede depois de correr*” and “*Carlos abriu a porta da geladeira*”, followed by the sentence “Write here in one sentence what will happen next”.

In the Story Recall subtest, the proper name “Kicheol” was replaced with the proper name “*José*”. Additionally, “Seoul station” was replaced with “*Aeroporto de Aracaju*” (Aracaju airport). Aracaju is the capital of the state of Sergipe, where Itabaianinha – the city where the population for whom the instrument was translated and adapted resides – is located. “Metro Station” was replaced with “*rodoviária*” (bus station) since our region (as well as several locations in Brazil) has no train stations or subways. “Red pepper paste” was replaced with “*molho de tomate*” (tomato sauce), which is a condiment better known in the region. “Middle school” was replaced with “*Ensino fundamental*” (elementary school) to align with the Brazilian education system level.

In the calculation test, we replaced the Korean currency *Won* with the Brazilian currency *Real*. In the step Story Recall, the proper name “Cheolsoo” was replaced with *Pedro* (Peter)*.* The answers to the question “How did she carry her objects?” were translated and adapted to *equilibrando na cabeça* (balancing on her head), *uma estava na cabeça e outra nas mãos* (one was on her head and the other was in her hands), and “is not mentioned in the story” was replaced with *não é mencionado na história*.

In Color and Object Recognition, eight items that were unfamiliar to the target population were replaced. This change maintained the nature of the objects, with animals being replaced with animals and plants with plants. Thus, the following replacements were made: “white radish” with *cebolinha* (spring onion), “walnut” with *abacate (*avocado), “perilla leaf” with *coentro* (coriander), “lotus root” with *abacaxi* (pineapple), “cucumber” with *tomate* (tomato), “chili pepper” with *pimenta vermelha* (red pepper), “squirrel” with *rato* (rat), and “goldfish” with *camarão* (shrimp). All the replacement items are well known in the region.

The instrument’s application duration was 39 ± 8.6 minutes among (normal) literate individuals and 48.5 ± 5.8 minutes among illiterate individuals. Cronbach’s alpha based on all standardized items of the questionnaire was 0.76, indicating good reliability of the instrument. The interobserver and intraobserver variabilities were 99% and 96%, respectively.

The Portuguese version of the LICA instrument is presented in Supplementary Material 1, and the images used in the Color and Object Recognition and Naming subtest are presented in Supplementary Material 2.

## DISCUSSION

We presented herein the Portuguese version of the LICA instrument, which was translated into Brazilian Portuguese and culturally adapted for Brazil (7,18-20). The purpose of this version is to assess the cognitive function of older individuals who are illiterate and are living in Northeastern Brazil. Developed in South Korea, the LICA instrument can be applied universally if translated into local languages and culturally adapted to the local environment. The version of the instrument presented herein was created from a specific need to evaluate the cognitive performance of older individuals with lifelong, congenital, untreated IGHD (23-27). After the translation and cultural adaptation of this instrument, we are convinced of its usefulness in evaluating dementia disorders at any stage in Brazil and, after minor adaptations, in other Portuguese-speaking countries with high illiteracy rates (1). The inverse relationship between schooling and dementia is unquestionable (2-5). In Brazil, with an illiteracy rate of 11.2%, it is estimated that more than 1 million people have dementia, of whom 77% are undiagnosed. In 2016, dementia ranked second among the leading causes of death in people aged ≥ 70 years (8) and was the second and third cause of disability among older women and men, respectively (30). This represents enormous challenges for public health and care of older individuals in Brazil and other Portuguese-speaking countries.

The translation of questionnaires or clinical and research instruments into Portuguese is much needed in Portuguese-speaking countries. We have previously used a Portuguese version of the Disabilities of the Arm, Shoulder, and Hand (DASH) questionnaire (31) to assess shoulder function in individuals with congenital IGHD (32). We also carried out the translation into Portuguese of the Diagnostic-Oriented Screening Scale for Anxiety Disorders: The Center for Epidemiological Studies Anxiety Scale (CESA), a useful tool for general anxiety screening and for common anxiety disorders (33). The experience of using and translating instruments in internal medicine, psychiatry, and psychology has motivated us to overcome the challenge of translating the LICA instrument into Portuguese. For this, we used a transdisciplinary approach, as currently recommended in health research (27,28).

Brazil and South Korea differ in many aspects. Located in South America, Brazil was discovered in 1500 by Portuguese explorers, who established their language and religion in the new colony. Currently, almost 70% of the Brazilian population is Catholic, while approximately 20% is Protestant. Located in Southeast Asia, the country of South Korea originated from the division of the territory of the former Korea after World War II (1945). Its official language is Korean, with almost half of the population having no religion, while 22.8% are Buddhists, 18.3% are Protestants, and nearly 10% are Catholics. In 2020, the South Korean GDP was USD 1.8 trillion (for a population of 51 million inhabitants in an area of almost 100,000 km^2^), which is slightly higher than the Brazilian GDP of USD 1.6 trillion (for a population of 213 million, in an area of 8.5 million km^2^). The Brazilian population is ethnically heterogeneous and combines the characteristics of European settlers (white), Africans (black), and native people (indigenous), while Korea has one of the most homogeneous populations in terms of ethnicity and linguistics in the world. The Brazilian currency is the *Real*, while the South Korean currency is the *Won*. All in all, the differences between the two countries in terms of fauna, flora, customs, food, and housing justify an adaptation of the Korean version of the LICA instrument into the one presented in this article. Small adaptations may be necessary in other Portuguese-speaking countries for the use of the Portuguese version presented here. The Brazilian Portuguese version of LICA was successfully applied 59 times and exhibited minimal variability and good reliability. The application duration was longer with the Portuguese version than with the Korean version (18), probably due to the respective lexicons, which are more concise in the latter language. The LICA instrument has been previously shown to identify improved attention and executive function in individuals with IGHD aged 50 years or older relative to GH-sufficient controls (34).

A potential limitation of the present study was the requirement of three levels of translation, from Korean to English and from English to Portuguese. Of note, the version that we translated into Portuguese was the English version of the previously published and validated South Korean LICA model (18-20); we adapted it to our local culture to evaluate the cognitive function of people with little or no formal education solely for the purpose of research. To achieve this objective, we used Beaton’s guidelines for the process of cross-cultural adaptation of self-report measures (29).

The Portuguese version of LICA was a valuable tool for the cognitive assessment of individuals with Itabaianinha syndrome. Born from an endocrinological research question, it appears to facilitate cognitive research in any area of research in Brazil and other Portuguese-speaking countries.
