# Supplementary material for: Portuguese version of the Literacy Independent Cognitive Assessment (LICA) instrument in the evaluation of individuals aged 50 years or older with Itabaianinha syndrome
Source: Arch Endocrinol Metab. 2024 Aug 13;68:e230265. doi: 10.20945/2359-4292-2023-0265 (PMC11460966; doi:10.20945/2359-4292-2023-0265)
Supplement: Supplementary file 1 [file 2359-4292-aem-68-e230265-suppl01.pdf]

Supplementary Material 1. The Literacy Independent Cognitive Assessment (LICA) instrument, Portuguese version

Avaliação cognitiva não dependente da alfabetização
(*Literacy Independent Cognitive Assessment* – LICA)

Faixa Etária 60-90 anos

Nome		N. do paciente	
Data de nascimento (Idade)		Setor de avaliação	
Gênero	( ) Masculino<br>( ) Feminino<br>( ) Outro	Profissional requisitante	
Escolaridade (número de anos)		Data de avaliação	
Domínio motor	( ) Destro<br>( ) Canhoto<br>( ) Ambidestro	N. da avaliação	
Cuidador	( ) Convive<br>( ) Não convive	Examinador	

NOTAS

Copyright© AE&M all rights reserved.

Avaliação da Alfabetização (S)

A avaliação da alfabetização testa a capacidade de leitura e escrita do paciente antes da doença (original) e a atual, e assim determina o seu nível de alfabetização. Consiste em um relato do cuidador e uma avaliação do paciente, ambos devem ser conduzidos por um terceiro. O mesmo critério de pontuação se aplica a ambas as avaliações.

1. Relato do Cuidador

Método

Pergunte ao cuidador sobre as habilidades de leitura e escrita do paciente antes da doença (original).

Instruções

Escrita: **"Antes da doença, ele/ela conseguia escrever suas próprias frases?"**
(**Sim:** 3 pontos, **Não:** faça a pergunta a seguir)
"Mas ele/ela conseguia escrever palavras?" (**Sim:** 2 pontos, **Não:** 1 ponto)

Leitura: **"Antes da doença, ele/ela conseguia ler e entender frases sem ajuda?**
(**Sim:** 3 pontos, **Não:** faça a pergunta a seguir)
"Mas ele/ela conseguia ler e entender palavras? (**Sim:** 2 pontos, **Não:** 1 ponto)

2. Avaliação do Paciente

Método

Oriente o paciente a ler as sentenças da página 3 e escrever uma frase descrevendo a situação a seguir. Se o paciente tiver sucesso na leitura e escrita das frases, termine a avaliação. Caso não seja bem-sucedido, execute a leitura e a escrita das palavras.

Instruções

Frases: (Mostrando a página 3.) "**Leia as seguintes frases na ordem que está".** (Se o paciente ler as duas sentenças, apontando para o espaço abaixo) **"Escreva aqui em uma frase o que vai acontecer depois".**

Palavras: (Se o paciente não ler com precisão pelo menos uma das frases, execute a leitura das palavras. Aponte para cada uma das palavras escritas em negrito na página 3, **muita, correr, geladeira**) **"Tente ler esta palavra".** (Independentemente de o paciente ser bem-sucedido, execute a escrita das palavras) **"Escreva as palavras que eu vou ler em voz alta".** (Leia em voz alta as seguintes palavras: **cachorro, foto, música**)

Pontuação (aplica-se tanto à avaliação do paciente como ao relato do cuidador)

Nível	Pontuação	Descrição
**Insuficiente**	1	Falha
**Palavras**	2	Sucesso na leitura e escrita de palavras (as três palavras)
**Frase**	3	Sucesso na leitura e escrita de frases (leitura de frases e escrita)

- A escrita de frases é bem-sucedida se a frase contiver duas ou mais palavras e puder ser entendida independentemente da ortografia.
- A leitura e escrita de palavras é bem-sucedida se todas as três palavras forem lidas e escritas com precisão (ortografia correta é necessária).

Resumo da Pontuação da Avaliação de Alfabetização (Inserir a pontuação)

	**Relato do cuidador sobre as habilidades antes da doença**		**Avaliação do Paciente**	
	Escrita	Leitura	Escrita	Leitura
Pontuação				

Definição de Alfabetizado/Analfabeto

**Alfabetizado**	Se os resultados de leitura e escrita do paciente forem todos 3, ou se os resultados de leitura e escrita do relatório do cuidador forem todos 3
**Analfabeto**	Todos os outros casos

Copyright© AE&M all rights reserved.

Carlos estava com **muita** sede depois de **correr**.

↓

Carlos abriu a porta da **geladeira**

↓

Escrita da Frase
**Escrita de Palavras**

Copyright© AE&M all rights reserved.

1. Memorizando uma História – Memória Imediata

Método

• Conte uma pequena história com um tema específico e peça ao paciente que a relembre imediatamente.

Pontuação

Registrar as respostas do paciente na seção "Resposta (conteúdo relatado)". Se a resposta corresponder à história, marcar com um "V", caso não corresponda, registrar a resposta exatamente como foi dita e pontuar de acordo com o critério de pontuação na página seguinte.

1 ponto: se a resposta corresponder ao original/se o sentido for o mesmo como no exemplo da página 5
0,5 ponto: se o sentido for parcialmente semelhante

Instruções

"Agora eu vou lhe contar uma pequena história, chamada "A história do estudante que ajudou a idosa". "Ouça com atenção e tente se lembrar, depois me conte a história da maneira mais parecida possível". (Deixe uma pausa clara nos lugares marcados /, e faça uma pausa de 2 segundos no intervalo entre as sentenças).

José / um estudante do ensino médio / encontrou / uma / mulher idosa / em frente ao aeroporto de Aracaju. (Intervalo de 2 segundos)

A mulher idosa / estava indo/ para a casa / da filha dela / levando / uma caixa / de ovos / e um frasco / de molho de tomate. (Intervalo de 2 segundos)

José / levou / o frasco / de molho de tomate / para a rodoviária. (Intervalo de 2 segundos)

"Agora me conte a história que acabei de contar, o melhor que você puder".

Item	História	Resposta (conteúdo relatado)	Pontuação		
1	José		0	0,5	1
2	Um estudante do ensino médio		0	0,5	1
3	Encontrou		0	0,5	1
4	Uma		0	0,5	1
5	Mulher idosa		0	0,5	1
6	Em frente ao aeroporto de Aracaju		0	0,5	1
7	A mulher idosa		0	0,5	1
8	Estava indo		0	0,5	1
9	Para a casa		0	0,5	1
10	Da filha dela		0	0,5	1
11	Levando		0	0,5	1
12	Uma caixa		0	0,5	1
13	De ovos		0	0,5	1
14	E um frasco		0	0,5	1
15	de molho de tomate		0	0,5	1
16	José		0	0,5	1
17	Levou		0	0,5	1
18	O frasco		0	0,5	1
19	De molho de tomate		0	0,5	1
20	Para a rodoviária		0	0,5	1
			Pontuação Total: ___/20		

Copyright© AE&M all rights reserved.

1. Memorizando uma História – Memória Imediata (continuação)

Pontuação
• Seguir os seguintes critérios de pontuação e exemplos.

História	Critério de pontuação	0 ponto (exemplo)	0,5 ponto (exemplo)	1 ponto (exemplo)
José	Deve ser "**José"**	Carlos/João		José
Um estudante do ensino médio	Deve ser "**ensino médio"**	Ensino fundamental		Ensino médio (estudante)
Encontrou	Deve significar **"encontrou"**		Viu/Avistou	Encontrou
Uma	Deve significar **"uma paciente"**	Duas pacientes		Uma (paciente)
Mulher idosa	Deve significar **"mulher idosa"**	Homem idoso/Vovô	Paciente idosa	Mulher idosa/Mulher velha
Em frente ao aeroporto de Aracaju	Deve ser "**aeroporto de Aracaju**"	Rodoviária de Aracaju/Aeroporto de São Paulo	No aeroporto/Em frente ao aeroporto	No aeroporto de Aracaju/Em frente ao aeroporto de Aracaju
A mulher idosa	Deve significar **"mulher idosa"**	Homem idoso/Vovô	Paciente idosa	Mulher idosa/Mulher velha
Estava indo	Deve significar "**estava indo**"	Foi	Estava pensando em ir/Estava planejando ir/Estava para chegar	Estava indo
Para a casa	Deve significar "para a **casa**"	Para o *shopping*	Para o apartamento	Para a casa
Da filha dela	Deve significar "**filha**"	Neta/Sobrinha	Parente	Da filha dela/Filha
Levando	Deve significar "**levando**"	Levando na cabeça		Levando/Carregando/ Transportando
Uma caixa	Deve que significar "**caixa**"		Um pacote/Um punhado/Uma dúzia	Uma caixa
De ovos	Deve significar "**ovos**"	Bola		Ovos
E um frasco	Deve significar "**um** frasco"	Caixa/Sacola		Pote/Compota/Frasco
de molho de tomate	Deve ser "**molho de tomate**"	Molho vinagrete/ Molho de alho		De molho de tomate
José	Deve ser "**José**"	Carlos/Antônio		José
Levou	Deve significar "**levou**"	Pegou o frasco		Levou, carregou, entregou
O frasco	Deve significar "**um** frasco"	Caixa/Sacola		Pote/Compota/Frasco
De molho de tomate	Deve ser "**molho de tomate**"	Molho vinagrete/ Molho de alho		De molho de tomate
Para a rodoviária	Deve significar "**Rodoviária**"	Ponto de táxi		Até a rodoviária/ Ponto de ônibus

Copyright© AE&M all rights reserved.

2. Montagem com Palitos (S1)	
**Material**<br>• **4 palitos, Quadro de Figuras**	
**Método**<br>• O paciente usa 4 palitos, cada um com uma ponta colorida de vermelho, para reproduzir as formas apresentadas por meio de fotos. Há duas questões de treino e dez para a avaliação. Não há limite de tempo. As formas apresentadas durante essa atividade são estímulos para o teste de Reconhecimento Visual, realizado posteriormente na avaliação. Portanto, após cada pergunta, o paciente deve ser instruído a memorizar a forma.	
**Pontuação**<br>• **1 ponto:** Tanto a forma quanto a posição das pontas vermelhas estão corretas<br>• **0,5 ponto:** A forma é correta, mas as pontas vermelhas estão posicionadas incorretamente<br>• **0 ponto:** A forma é incorreta	
**Instruções**	
**Questão Treino**	
(Mostrando os palitos) "Aqui estão quatro palitos, que têm pontas coloridas de vermelho. Vou lhe mostrar uma foto e você vai fazer o mesmo desenho usando estes palitos. Coloque as pontas vermelhas também na mesma posição. Primeiro, vamos treinar".<br><br>"Coloque os palitos na mesma posição que você está vendo nessa foto. Coloque as pontas vermelhas também na mesma posição".	
**Se a resposta está correta:**	**"Muito bem, é assim que é para fazer".**
**Se a resposta está errada:**	**"Olhe com mais atenção. Está igual a foto?"**<br>(Então dê mais uma chance).<br><br>Se o paciente não conseguir reproduzir a forma na segunda tentativa, o examinador deve montar a imagem e explicar. Em seguida, passe para a segunda questão de treino ou para as perguntas do teste.
**Questão teste**	
"Coloque os palitos na mesma posição que você está vendo nessa foto. Coloque as pontas vermelhas também na mesma posição".	
**Se a resposta está correta:**	**"Muito bem, é assim que é para fazer".**
**Se a resposta está errada:**	**"Olhe com mais atenção. Está igual a foto?"**<br>(Então dê mais uma chance).
(Quando a questão estiver completa, independentemente de a resposta ter sido correta ou incorreta)<br>**"Tente se lembrar desta foto. Vou perguntar sobre ela mais tarde".**<br><br>(Mostre a imagem por 5 segundos, depois passe para a próxima questão).	

Copyright© AE&M all rights reserved.

2. Montagem com Palitos (S1) (continuação)					
**Questão**	**Estímulo**	**Para pontuar**	**Resposta (se a pontuação não for 1, desenhe a forma)**	**Pontuação**	
Questão Treino 1	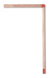	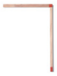			
Questão Treino 2	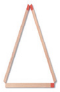	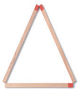			
1	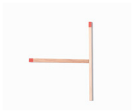	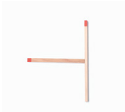		0	0,5 1
2	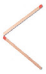	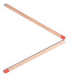		0	0,5 1
3	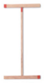	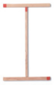		0	0,5 1
4	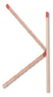	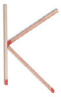		0	0,5 1
5	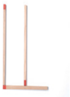	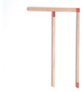		0	0,5 1
6	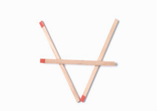	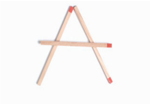		0	0,5 1
7	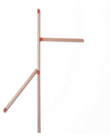	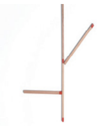		0	0,5 1
8	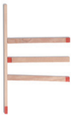	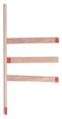		0	0,5 1
9	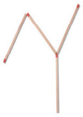	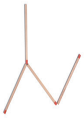		0	0,5 1
10	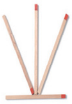	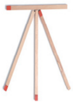		0	0,5 1
(Não pontuar as questões treino) Pontuação Total: ___/10					

Copyright© AE&M all rights reserved.

3. Memorizando Palavras – Memória Imediata (S2)

Método

Leia claramente 10 palavras, uma palavra a cada dois segundos. Depois, oriente o paciente a lembrar o maior número de palavras que puder. Repita o teste três vezes, lendo as dez palavras na mesma ordem. Instrua o paciente a falar novamente as palavras ditas nas rodadas anteriores. Se o paciente responder de forma negativa ou sem confiança, encoraje-o o suficiente para obter uma resposta. Não oriente o paciente a memorizar as palavras no final da terceira rodada.

Pontuação

- Enumere a ordem na qual as respostas são dadas.
- Anote quaisquer palavras que não estejam listadas na seção “Respostas Incorretas”.
- Toda resposta correta recebe um ponto.

Instruções

Primeira Rodada: **“Daqui a pouco vou ler algumas palavras. Escute com atenção. Quando eu terminar de falar, repita o maior número de palavras que você puder lembrar. Não se preocupe com a ordem das palavras. Agora, eu vou ler as palavras”.** (depois de ler todas as palavras) **“Agora, fale o maior número de palavras você consegue lembrar”.** (se for necessário motivar) **“Você consegue lembrar mais alguma coisa?”**

Rodadas 2 e 3: **“Vou ler as palavras que você acabou de ouvir de novo. Mais uma vez, tente se lembrar do maior número de palavras que puder me dizer, independentemente da ordem. Você deve dizer de novo as palavras que você já disse nas rodadas anteriores. Agora, eu vou ler as palavras”.**

(depois de ler todas as palavras) **“Agora, fale o maior número de palavras que você consegue lembrar”.** (se for necessário motivar) **“Você consegue lembrar mais alguma coisa?”**

		Rodada 1			Rodada 2			Rodada 3		
**Nº**	**Item**	**Ordem de resposta**	**Pontuação**		**Ordem de resposta**	**Pontuação**		**Ordem de resposta**	**Pontuação**	
1	Couve		0	1		0	1		0	1
2	Martelo		0	1		0	1		0	1
3	Pepino		0	1		0	1		0	1
4	Meia		0	1		0	1		0	1
5	Serrote		0	1		0	1		0	1
6	Luva		0	1		0	1		0	1
7	Feijão		0	1		0	1		0	1
8	Cebola		0	1		0	1		0	1
9	Tesoura		0	1		0	1		0	1
10	Saia		0	1		0	1		0	1
Respostas erradas (palavras que não estão na lista)										
		Pontuação: ___/10			Pontuação: ___/10			Pontuação: ___/10		

Copyright© AE&M all rights reserved.

4. Visão Espacial

Material
• Tábua de teste da Visão Espacial

Método
• Sente-se de frente para o paciente, com o lado numerado da placa do teste de Visão Espacial voltado para o examinador. Execute o teste “na ordem direta” e depois “na ordem inversa”. Primeiro, o examinador aponta para os blocos, um a cada segundo, e depois o paciente aponta para os blocos na mesma ordem ou em ordem inversa. Para cada número de blocos, se o paciente responder com sucesso a primeira tentativa, considere a segunda também correta e passe para o próximo número de blocos. Se o paciente falhar na primeira tentativa, passe para a segunda tentativa. Se o paciente falhar em ambas as tentativas, interrompa o teste.

Pontuação
• Assinalar “O” na coluna Resultados para uma resposta correta, e se o paciente der uma resposta incorreta, assinalar “X” e registrar a resposta.
A pontuação do teste é o maior número de blocos que o paciente tentou com sucesso pelo menos uma vez.

4-1) Ordem direta

Instruções

“Tenho alguns blocos aqui. Vou apontar para os blocos em uma determinada ordem, e então você vai repetir depois de mim na mesma ordem. Primeiro, vamos treinar”. (Depois de apontar para 1, 4) **“Aponte para os blocos nessa mesma ordem que eu”.**

**Se a resposta estiver correta:**	**“Muito bem. Agora, eu vou começar o teste”.**
**Se a resposta estiver errada:**	(Apontando para 1 e depois 4) **“Eu apontei para os blocos nesta ordem, então você também deveria apontar nessa mesma ordem que eu”.** (apontar para 1 e depois 4)<br><br>(Apontando para 7 e depois 2) **“Aponte para os blocos na mesma ordem que eu”.** (Se o paciente fizer errado novamente, mostre a resposta e continue o teste)<br><br>**“Agora, vou começar o teste. Observe com atenção e repita depois de mim”.**

Número de Blocos	Tentativa	Gabarito	Resposta	Resultado	
2	1	6-5		O	X
	2	3-8		O	X
3	1	7-2-9		O	X
	2	8-5-4		O	X
4	1	6-1-7-2		O	X
	2	3-9-8-4		O	X
5	1	1-6-3-5-4		O	X
	2	2-7-6-1-9		O	X
6	1	8-7-3-9-4-5		O	X
	2	5-1-6-7-2-8		O	X
7	1	3-9-8-5-4-1-7		O	X
	2	4-1-8-3-9-2-5		O	X
8	1	7-2-5-8-1-9-4-6		O	X
	2	9-1-4-7-6-3-2-5		O	X
					Pontuação: ___/8

Copyright© AE&M all rights reserved.

4. Teste de Visão Espacial (continuação)

4-2) Ordem Inversa

Instruções

"Agora, aponte para os blocos na ordem contrária. Primeiro, vamos treinar". (Depois de apontar para 1, 4) **"Aponte para os blocos na ordem contrária".**

**Se a resposta estiver correta:**	**"Muito bem. Agora, eu vou começar o teste".**
**Se a resposta estiver errada:**	**"Preste atenção.** (apontando para 1 e depois 4) **Eu apontei para os blocos nesta ordem, então você deveria** (apontando para 4 e depois 1) **apontar na ordem contrária, como estou fazendo agora.** (apontando para 7 e depois 1) **Aponte os blocos na ordem contrária, de trás pra frente".** (Se o paciente fizer errado novamente, mostre a resposta e continue o teste)<br>**"Agora, vou começar o teste. Observe com atenção e repita na ordem contrária".**

Número de Blocos	Tentativa	Ordem do Examinador (Ordem do Gabarito)	Resposta	Resultado	
2	1	**8-5**<br>(5-8)		O	X
	2	**7-2**<br>(2-7)		O	X
3	1	**3-9-8**<br>(8-9-3)		O	X
	2	**6-1-7**<br>(7-1-6)		O	X
4	1	**2-7-6-1**<br>(1-6-7-2)		O	X
	2	**1-6-3-5**<br>(5-3-6-1)		O	X
5	1	**5-1-6-7-2**<br>(2-7-6-1-5)		O	X
	2	**8-7-3-9-4**<br>(4-9-3-7-8)		O	X
6	1	**4-1-8-3-9-2**<br>(2-9-3-8-1-4)		O	X
	2	**3-9-8-5-4-1**<br>(1-4-5-8-9-3)		O	X
7	1	**9-1-4-7-6-3-2**<br>(2-3-6-7-4-1-9)		O	X
	2	**7-2-5-8-1-9-4**<br>(4-9-1-8-5-2-1)		O	X
8	1	**3-8-5-4-9-2-7-6**<br>(6-7-2-9-4-5-8-3)		O	X
	2	**6-5-7-2-9-1-3-4**<br>(4-3-1-9-2-7-5-6)		O	X
					Pontuação: ___/8

Copyright© AE&M all rights reserved.

5. Teste *Stroop* com Números (S3)

Material
• Ficha do Teste *Stroop* com números, cronômetro

Método
• O teste *Stroop* tem duas partes. Durante a Parte 1, o paciente lê o número escrito dentro de cada célula, e na Parte 2, soma a quantidade de dígitos escritos em cada célula. Faça uma pergunta de treino antes do teste usando a linha de fundo da “placa de estímulo”. Faça a Parte 1 e depois a Parte 2. O tempo limite é de 3 minutos para cada parte.

Pontuação
• Registre o número de respostas corretas e respostas incorretas.
• Se o paciente corrige sua resposta para dar a resposta correta, registre-a como correta.
• Registre o tempo gasto para a tarefa, incluindo o tempo usado para corrigir quaisquer respostas. Se a tarefa não for concluída em 3 minutos, pare o teste. As células que não foram tentadas não serão incluídas no número de respostas corretas ou incorretas.
• Repita as instruções abaixo quantas vezes forem necessárias e realize os treinos para garantir que o paciente compreenda totalmente a tarefa. Em seguida, prossiga com o teste, observe e registre o número de respostas corretas dadas durante os 3 minutos.

Instruções

Parte 1:
“Vamos olhar para a última linha. Há números escritos em cada quadradinho. Leia o número que aparece em cada quadradinho apenas uma vez, como 2, 1, 3. Vamos tentar”. (Se o paciente ler 2/1/3/2/1) “Muito bem. Agora, quando eu falar ‘comece’, leia o número escrito em cada quadradinho o mais correto e rápido que puder, daqui até o final. (apontando transversalmente na placa). Comece”.

Parte 2:
“Desta vez, me diga a quantidade de números escritos dentro de cada quadradinho. Se houver apenas um número dentro, diga um. Se tiver dois números, diga dois, e assim por diante”.
(se o paciente falar 2/1/2/3/1) “Muito bem. Agora, quando eu falar ‘comece’, conte a quantidade de números em cada quadradinho o mais correto e rápido que puder, daqui até o final (apontando transversalmente na placa). Comece”.

Parte 1 Resposta						Parte 2 Resposta					
	2	1	3	2	1		1	2	1	3	2
	2	3	1	3	1		3	2	1	3	3
	1	3	3	2	2		3	1	2	2	3
	1	2	3	1	2		1	2	3	1	2
	3	2	3	3	1		3	1	2	3	1
	2	3	2	1	3		3	2	1	3	2
	2	3	1	2	2		2	3	1	2	1
	1	3	1	1	3		1	1	2	3	3
	3	2	3	2	1		2	2	1	3	3
	2	1	3	2	1		2	1	2	3	1

	Respostas corretas	Respostas erradas	Tempo
Parte 1	/50	/50	Segundos
Parte 2	/50	/50	Segundos

Copyright© AE&M all rights reserved.

6. Cálculo

Método

• O paciente realiza tarefas simples de cálculo sem utilizar caneta e papel. O teste utiliza cálculos monetários simples para avaliar habilidades de adição, subtração, multiplicação e divisão. Conduza a tentativa 1 primeiro. Se a primeira tentativa for respondida com sucesso, pule a segunda tentativa e marque-a como correta. Se a resposta à primeira tentativa estiver incorreta, passe para a segunda tentativa e marque-a de acordo com as respostas do participante. Se o paciente errar na primeira e na segunda tentativa, interrompa o teste e passe para a próxima seção. Não há limite de tempo, e o paciente pode pedir ao examinador que leia novamente as perguntas.

Pontuação

•**1 ponto:** Resposta correta
•**0 ponto:** Resposta errada

Instruções

"Eu vou ler algumas questões simples de matemática. Ouça com atenção e me diga a resposta".

Seção	Tentativa 1	Pontuação		Tentativa 2	Pontuação		Subtotal
Adição	Quanto é 10 reais mais 30 reais? (40 reais)	0	1	Quanto é 10 reais mais 20 reais? (30 reais)	0	1	__/6
	Quanto é 50 reais mais 30 reais? (80 reais)	0	1	Quanto é 50 reais mais 20 reais? (70 reais)	0	1	
	Quanto é 70 reais mais 80 reais? (150 reais)	0	1	Quanto é 70 reais mais 50 reais? (120 reais)	0	1	
Subtração	Quanto é 30 reais menos 20 reais? (10 reais)	0	1	Quanto é 30 reais menos 10 reais? (20 reais)	0	1	__/6
	Quanto é 80 reais menos 40 reais? (40 reais)	0	1	Quanto é 80 reais menos 30 reais? (50 reais)	0	1	
	Quanto é 120 reais menos 30 reais? (90 reais)	0	1	Quanto é 120 reais menos 50 reais? (70 reais)	0	1	
Multiplicação	Se você guardar 100 reais todo dia, quanto você tem depois de 3 dias? (300 reais)	0	1	Se você guardar 100 reais todo dia, quanto você tem depois de 2 dias? (200 reais)	0	1	__/6
	Se você guardar 20 reais todo dia, quanto você tem depois de 3 dias? (60 reais)	0	1	Se você guardar 20 reais todo dia, quanto você tem depois de 2 dias? (40 reais)	0	1	
	Se você guardar 50 reais todo dia, quanto você tem depois de 5 dias? (250 reais)	0	1	Se você guardar 50 reais todo dia, quanto você tem depois de 3 dias? (150 reais)	0	1	
Divisão	Quantas notas de 10 reais você precisa para ter 40 reais? (4 notas)	0	1	Quantas notas de 10 reais você precisa para ter 30 reais? (3 notas)	0	1	__/6
	Quantas notas de 50 reais você precisa para ter 250 reais? (5 notas)	0	1	Quantas notas de 50 reais você precisa para ter 150 reais? (3 notas)	0	1	
	Quantas notas de 50 reais você precisa para ter 450 reais? (9 notas)	0	1	Quantas notas de 50 reais você precisa para ter 350 reais? (7 notas)	0	1	
							Pontuação Total __/24

Copyright© AE&M all rights reserved.

7. Memorizando uma História – Memória Tardia

Método

• Este teste é uma continuação do teste "Memorizando uma História – Memória Imediata". Dê ao paciente instruções para recordar a história que ouviu e memorizou anteriormente, sem dar nenhuma dica ou pista.

Pontuação

O método de pontuação é idêntico ao Memorizando uma História – Memória Imediata (Consulte a página 5, Pontuação).

Instruções

"Algum tempo atrás, eu contei para você 'A história do estudante que ajudou a idosa'. Conte-me a história do jeito que você se lembra".

Item	História	Resposta (conteúdo relatado)	Pontuação		
1	José		0	0,5	1
2	Um estudante do ensino médio		0	0,5	1
3	Encontrou		0	0,5	1
4	Uma		0	0,5	1
5	Mulher idosa		0	0,5	1
6	Em frente ao aeroporto de Aracaju		0	0,5	1
7	A mulher idosa		0	0,5	1
8	Estava indo		0	0,5	1
9	Para a casa		0	0,5	1
10	Da filha dela		0	0,5	1
11	Levando		0	0,5	1
12	Uma caixa		0	0,5	1
13	De ovos		0	0,5	1
14	E um frasco		0	0,5	1
15	De molho de tomate		0	0,5	1
16	José		0	0,5	1
17	Levou		0	0,5	1
18	O frasco		0	0,5	1
19	De molho de tomate		0	0,5	1
20	Para a rodoviária		0	0,5	1
		Pontuação Total: ___/20			

Copyright© AE&M all rights reserved.

8. Memorizando uma História – Reconhecimento

Método

- Conduza este teste imediatamente após "Memorizando uma História – Memória Tardia". Faça perguntas de múltipla escolha sobre "A história do estudante que ajudou a idosa" para verificar se o paciente se lembra da história.
- Oriente o paciente para escolher 1 das 3 opções dadas. Se o paciente responder à pergunta 3 incorretamente, diga a ele que "o personagem principal encontrou uma senhora idosa" antes de passar para a próxima pergunta.

Pontuação

- **1 ponto:** Resposta correta (caixa sombreada)
- **0 ponto:** Resposta incorreta

Instruções

"Vou fazer algumas perguntas sobre a história. Escolha a resposta certa".

Pergunta	Resposta		
1. Qual era o nome do personagem principal?	Pedro	Carlos	José
2. Qual o nível de escolaridade do estudante?	Ensino fundamental	Ensino médio	Ensino superior
3. Quem ele encontrou?	Homem idoso	Mulher idosa	Um homem
4. Onde ele encontrou a idosa?	Ponto de ônibus	No aeroporto de Aracaju	No aeroporto de Salvador
5. Com quantas mulheres idosas ele encontrou?	1	2	3
6. Qual desses objetos, a mulher idosa não estava carregando?	Ovos	Peixe	Molho de tomate
7. Como ela estava carregando as coisas dela?	Equilibrando na cabeça	Uma estava na cabeça e outra estava nas mãos	Não é mencionado na história
8. Para onde ela estava indo?	Para a casa do filho dela	Para a casa da filha dela	Para a escola do neto dela
9. Até onde ele acompanhou a idosa?	Estação de trem	Rodoviária	Restaurante
10. Dos objetos que ela estava carregando, qual foi o que ele levou para ela?	Ovos	Peixe	Molho de tomate
			Pontuação Total: __/10

Copyright© AE&M all rights reserved.

9. Reconhecimento Visual (S4)

Material
• Quadro do Teste de Reconhecimento Visual

Método
• Este teste é uma continuação do teste de “Montagem com Palitos”. Mostre 20 fotos uma a uma e oriente o paciente a dizer “Sim” caso a foto já tenha sido apresentada antes ou “Não” se não foi.

Pontuação
• **1 ponto:** Resposta correta (caixa sombreada)
• **0 ponto:** Resposta incorreta

Instruções

“Há algum tempo, você olhou algumas fotos e fez o mesmo desenho usando palitos. Agora, vou mostrar mais algumas fotos. Se você achar que a foto é igual à do exercício que você fez, diga ‘Sim’, se não, diga ‘Não’”..

Questão (condição)	Sim	Não
1 (IR)		
2 (IR)		
3 (C)		
4 (IR)		
5 (C)		
6 (C)		
7 (R)		
8 (IR)		
9 (C)		
10 (C)		
11 (R)		
12 (C)		
13 (R)		
14 (C)		
15 (R)		
16 (C)		
17 (IR)		
18 (C)		
19 (C)		
20 (R)		
	Respostas “Sim” (sombreados em “sim”) / 10	Respostas “Não” (sombreados em “não”) / 10

C: Correta (Resposta), R: Relacionado (Relacionado, mas incorreta), IR: Incorreta e não relacionada	Pontuação Total /20

Copyright© AE&M all rights reserved.

10. Memorizando Palavras – Memória Tardia (S5)

Método

• Este teste é uma continuação do teste "Memorizando Palavras – Memória Imediata". Instrua o paciente a recordar e relatar as palavras que memorizou sem nenhuma dica ou pista.

Pontuação

- Enumere a ordem na qual as respostas são dadas.
- Recorde quaisquer palavras que não estejam listadas na seção "Resposta incorreta".
- Todas as respostas corretas marcam um ponto cada.

Instruções

"Há um tempo, li uma lista de palavras. Diga para mim todas as palavras que você consegue lembrar da lista". (Se o paciente diz que não se lembra mais, encoraje-o) **"Você consegue lembrar mais alguma coisa?"**

Nº	Item	Ordem de respostas	Ponto	
1	Couve		0	1
2	Martelo		0	1
3	Pepino		0	1
4	Meia		0	1
5	Serrote		0	1
6	Luva		0	1
7	Feijão		0	1
8	Cebola		0	1
9	Tesoura		0	1
10	Saia		0	1
Respostas incorretas (palavras que não estão na lista)				
			Pontuação Total ___/10	

Copyright© AE&M all rights reserved.

11. Memorizando Palavras – Reconhecimento

Método

• Conduza este teste imediatamente após “Memorizando Palavras – Memória Tardia”. Leia uma lista de palavras, algumas das quais foram incluídas no “Memorizando palavras” e outras não, e faça o reconhecimento das palavras. Leia as palavras na ordem indicada e marque “X” na coluna “Sim” ou “Não” de acordo com a resposta do paciente.

Pontuação

1 ponto: Resposta correta (caixa sombreada)

Instruções

“Vou ler mais algumas palavras. Se for uma palavra que eu já disse antes, diga ‘Sim’, se não, diga ‘Não’”.

Nº	Item	Sim	Não	Nº	Item	Sim	Não
1	Couve (C)			11	Luva (C)		
2	Unha (R)			12	Feijão (C)		
3	Martelo (C)			13	Mesa (IR)		
4	Pepino (C)			14	Bicicleta (IR)		
5	Escada (IR)			15	Cebola (C)		
6	Alho (R)			16	Tesoura(C)		
7	Anel (IR)			17	Cigarro (IR)		
8	Meia (C)			18	Saia (C)		
9	Serrote (C)			19	Sacola (R)		
10	Pimenta (R)			20	Botas (R)		

Respostas “Sim” Corretas: ___/10
Respostas “Não” Corretas: ___/ 10

C: Correta (Resposta), R: Relacionado (Relacionado, mas incorreta), IR: Incorreta e não relacionada)	**Pontuação Total**<br><br>**Total de respostas corretas: / 20**

Copyright© AE&M all rights reserved.

12. Fluência Verbal (Animal) (S7)

Material
• Cronômetro

Método
• A tarefa é nomear o maior número possível de animais dentro de 1 minuto. Este teste avalia quantos nomes específicos pertencentes a uma categoria semântica o paciente pode relatar. O tempo limite é de um minuto, e se o paciente estiver em silêncio por mais de 15 segundos, repita as instruções e motive-o. Registre as respostas em intervalos de 15 segundos.

Pontuação
• Registre o número de animais aceitáveis.
• Serão excluídas as repetições e os nomes próprios.
• Se numa determinada espécie de animal houver denominações distintas para macho, fêmea ou filhote, eles são aceitos.
• O total da pontuação é o número total de nomes de animais aceitáveis que o paciente relatou por um minuto.

Instruções

"Diga para mim todos os nomes de animais que você consegue lembrar. Você terá um minuto para falar. Comece agora".

Tempo	Resposta	Número de Respostas
0~15 segundos		
16~30 segundos		
31~45 segundos		
46~60 segundos		
		**Pontuação Total:_______**

Copyright© AE&M all rights reserved.

13. Reconhecimento de Cores e Objetos/Nomeação (S8)

Material
• Quadro de Figuras

Método
• Uma imagem de um objeto comum é apresentada ao lado de uma versão modificada do objeto. O paciente deve escolher o objeto real e também nomeá-lo.

Pontuação
• Uma resposta correta em Reconhecimento de Cor e Objeto pontua 1 ponto.
• Uma resposta correta em Nomeação marca 1 ponto

Instruções

"Agora vou lhe mostrar fotos de frutas, legumes e animais. Uma é a foto do objeto real e a outra é uma versão modificada do objeto. Escolha a foto do objeto real e me diga o nome do objeto".

(mostrando a questão treino) "Primeiro, vamos fazer uma pergunta para você treinar. Escolha a foto real entre estas duas e me diga o que você está vendo".

(independentemente de o paciente estar correto) "A foto 1 é a real, e é um 'rabanete'. A tarefa é escolher a foto real e nomear o objeto. Se você estiver pronto, vou começar".

Questão	Gabarito/nome	Resposta (reconhecimento de cor e objeto)		Resposta (nomeação)		Resposta de nomeação incorreta
Questão Treino	1 / Cebolinha					
1	2 / Melancia	0	1	0	1	
2	1 / Tangerina ou laranja	0	1	0	1	
3	1 / Abacate	0	1	0	1	
4	2 /Cenoura	0	1	0	1	
5	2 / Coentro ou salsa	0	1	0	1	
6	2 / Abacaxi	0	1	0	1	
7	1 / Tomate	0	1	0	1	
8	1 / Pimenta	0	1	0	1	
9	2 / Amendoim	0	1	0	1	
10	1 / Cavalo	0	1	0	1	
11	2 / Coelho	0	1	0	1	
12	2 / Rato	0	1	0	1	
13	1 / Camarão	0	1	0	1	
14	1 / Porco	0	1	0	1	
15	1 / Pato	0	1	0	1	

Pontuação total de reconhecimento de cor e objeto:__/15
Pontuação total de nomeação__/15

Copyright© AE&M all rights reserved.

Supplementary Material 2. Images used in the Color and Object Recognition and Naming subtest

Reconhecimento de Cores e Objetos/Nomeação

Avaliação Cognitiva não dependente da alfabetização

(*Literacy Independent Cognitive Assessment* – LICA)

Referente à p. 19 do Formulário do Pesquisador

QUESTÃO TREINO

1

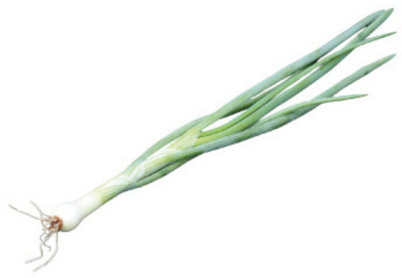

2

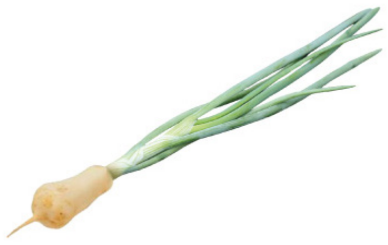

QUESTÃO 1

1

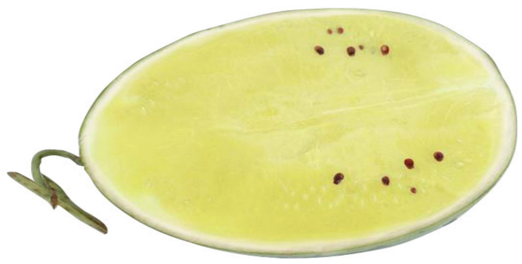

2

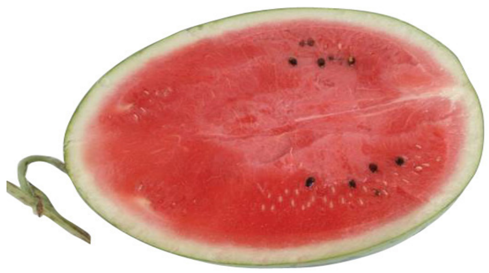

Copyright© AE&M all rights reserved.

QUESTÃO 2

1

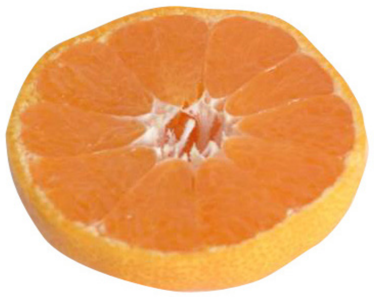

2

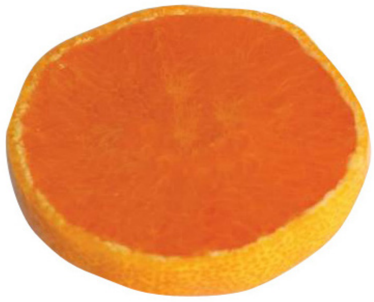

QUESTÃO 3

1

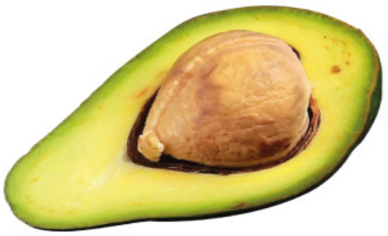

2

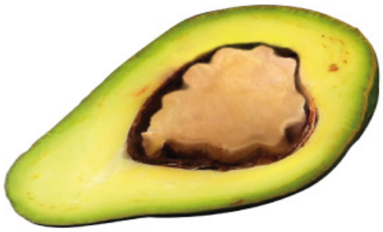

QUESTÃO 4

1

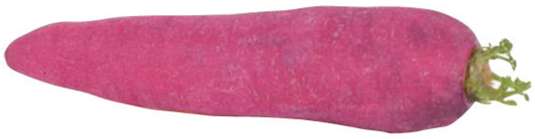

2

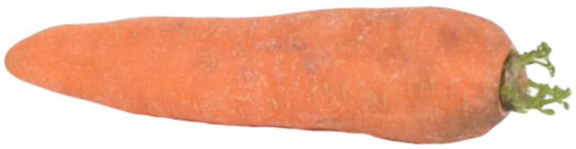

Copyright© AE&M all rights reserved.

QUESTÃO 5

1

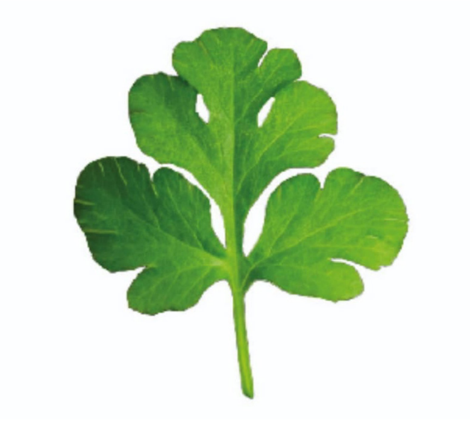

2

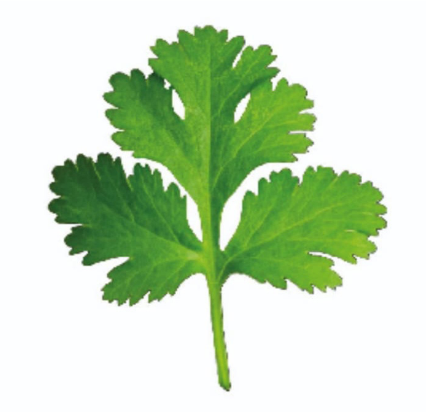

QUESTÃO 6

1

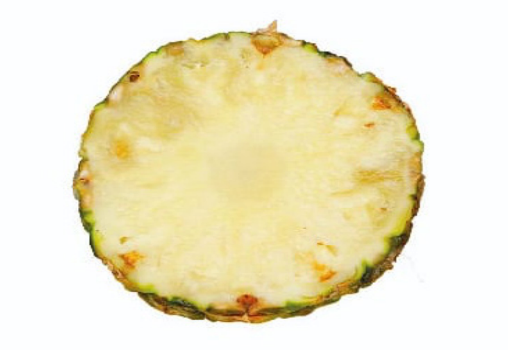

2

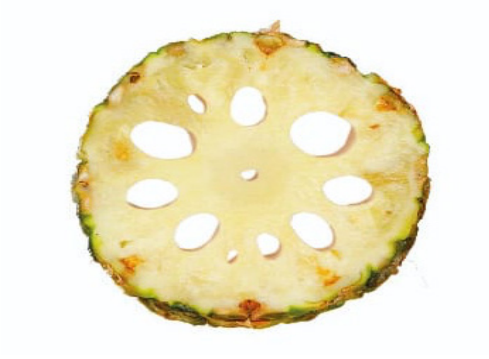

QUESTÃO 7

1

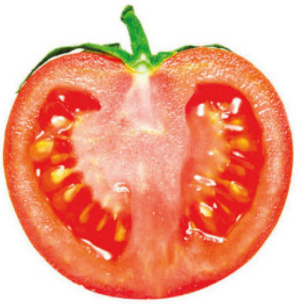

2

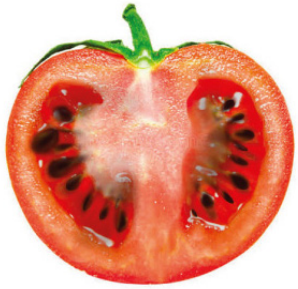

Copyright© AE&M all rights reserved.

QUESTÃO 8

1

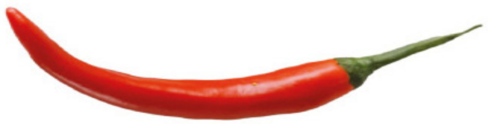

2

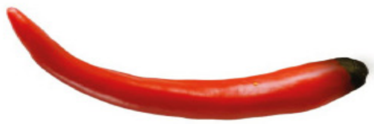

QUESTÃO 9

1

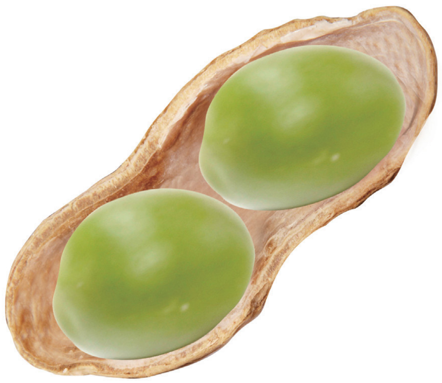

2

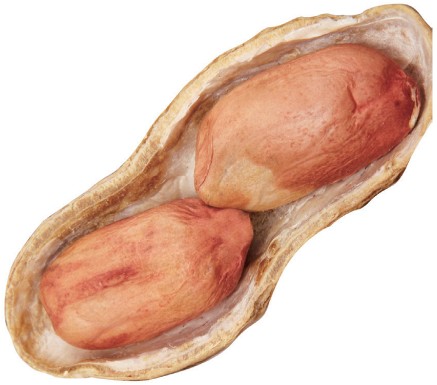

QUESTÃO 10

1

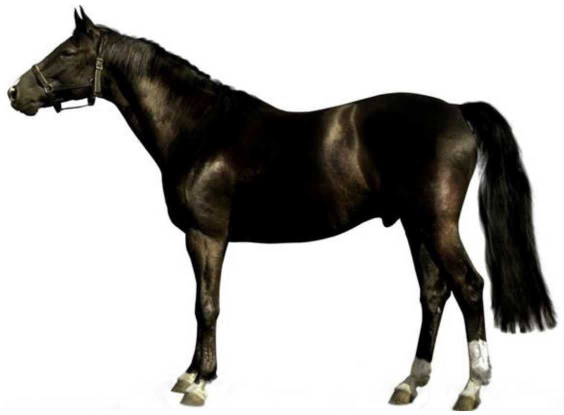

2

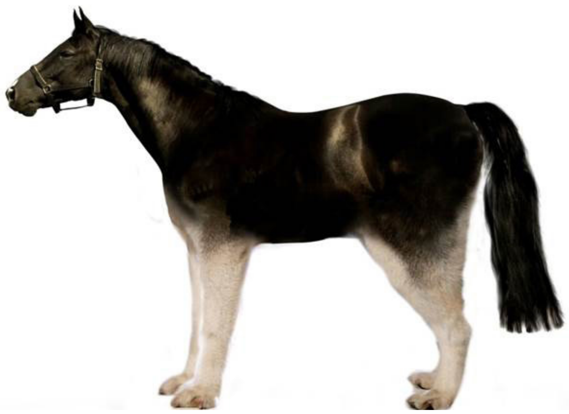

Copyright© AE&M all rights reserved.

QUESTÃO 11

1

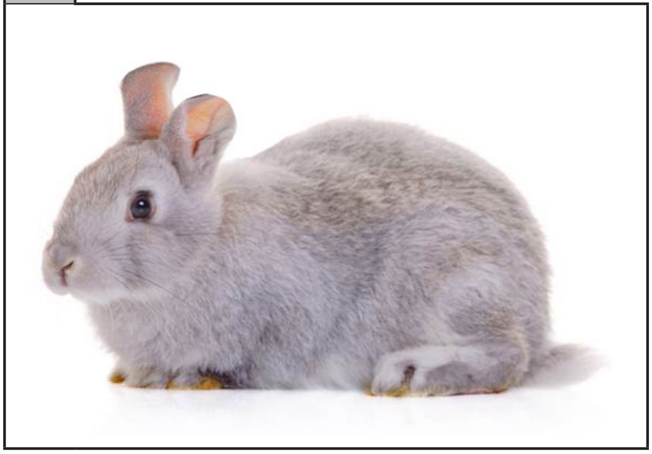

2

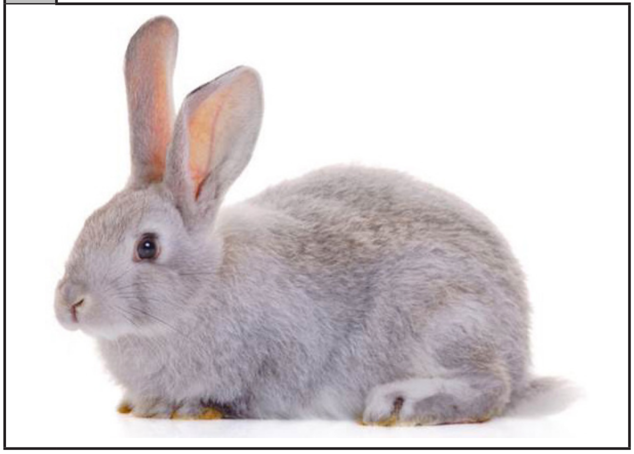

QUESTÃO 12

1

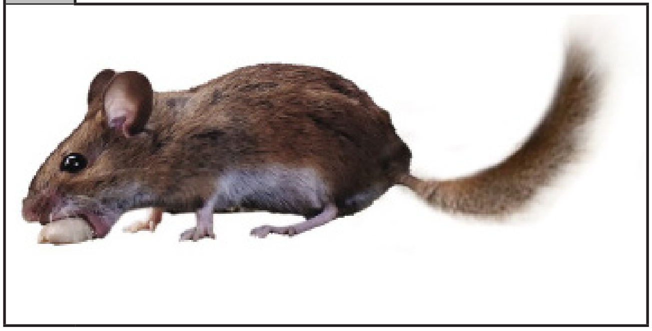

2

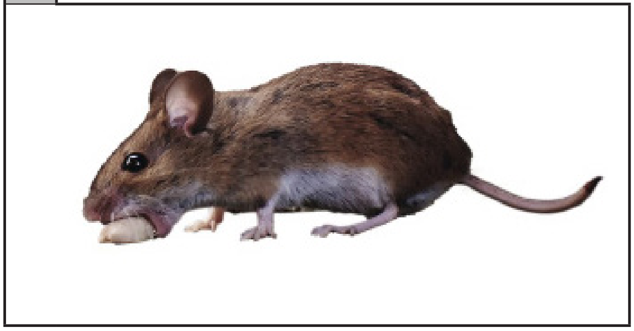

QUESTÃO 13

1

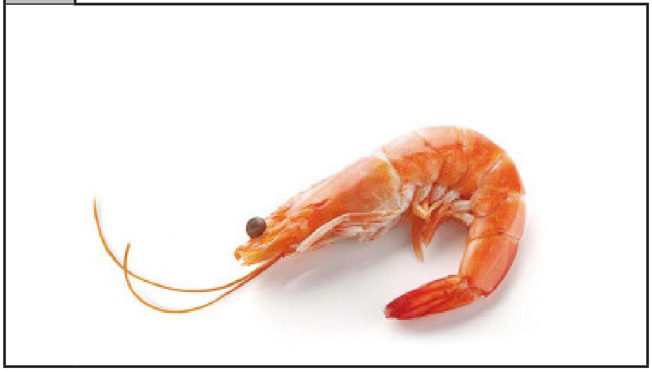

2

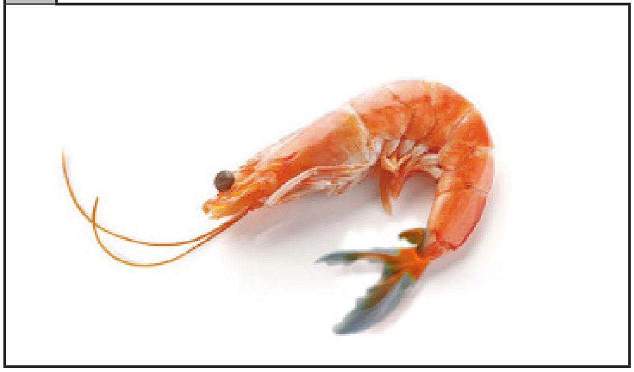

Copyright© AE&M all rights reserved.

QUESTÃO 14

1

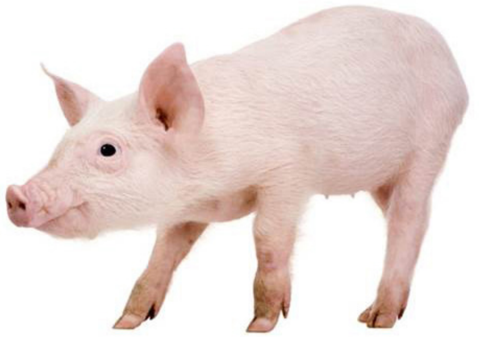

2

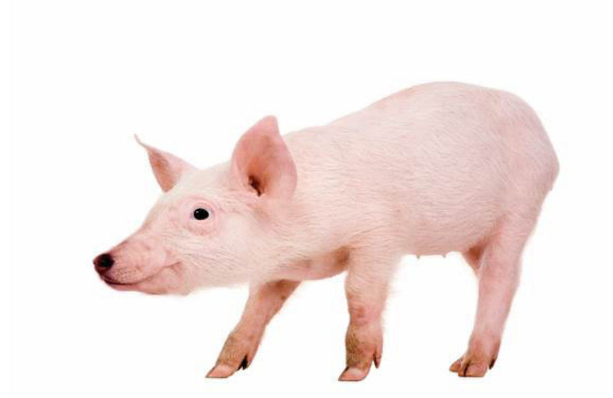

QUESTÃO 15

1

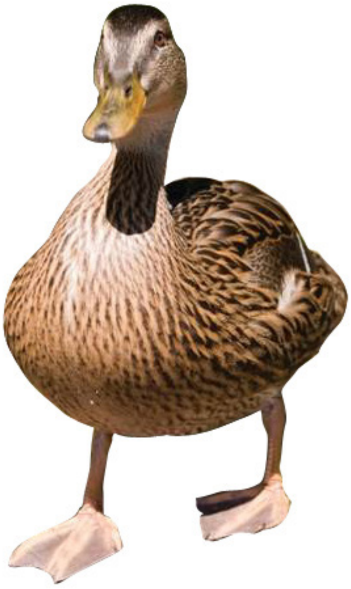

2

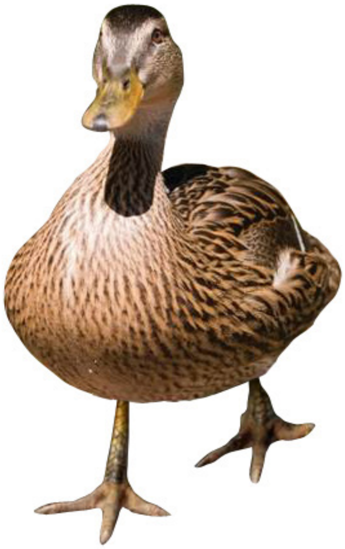

This is an open-access article distributed under the terms of the Creative Commons Attribution License

Copyright© AE&M all rights reserved.
